# Mitral valve prolapse morphofunctional features by cardiovascular magnetic resonance: more than just a valvular disease

**DOI:** 10.1186/s12968-021-00800-w

**Published:** 2021-10-11

**Authors:** Angélica Romero Daza, Aalap Chokshi, Patricia Pardo, Nicolas Maneiro, Ana Guijarro Contreras, Jose M. Larrañaga-Moreira, Borja Ibañez, Valentin Fuster, Leticia Fernández Friera, Jorge Solís, Javier Sanz

**Affiliations:** 1 Hospital La Luz-QuironSalud, Maestro Ángel Llorca, 8, 28003 Madrid, Spain; 2 Englewood Hospital and Medical Center, Englewood, NJ USA; 3 Department of Cardiology, Complexo Hospitalario Universitario A Coruña, A Coruña, Spain; 4 Hospital Universitario 12 de Octubre, Madrid, Spain; 5 Unidad de Gestión Clínica del Corazón, Hospital Clínico Universitario Virgen de la Victoria, Málaga, Spain; 6 Centro de Investigación Biomédica en Enfermedades Cardiovasculares, Instituto Biotecnológico de Málaga, Málaga, Spain; 7 Inherited Cardiovascular Diseases Unit, Cardiology Service, Complexo Hospitalario Universitario de A Coruña, Servizo Galego de Saúde (SERGAS), A Coruña, Spain; 8 Instituto de Investigación Biomédica de A Coruña (INIBIC), Universidad da Coruña, A Coruña, Spain; 9 Centro Nacional de Investigaciones Cardiovasculares Carlos III (CNIC), Madrid, Spain; 10 IIS-Fundación Jiménez Díaz Hospital, Madrid, Spain; 11 CIBERCV, Madrid, Spain; 12 Icahn School of Medicine at Mount Sinai, Zena and Michael A. Wiener Cardiovascular Institute, New York, NY USA; 13 Hospital Universitario HM Montepríncipe- CIEC, Madrid, Spain; 14 Universidad CEU San Pablo, Madrid, Spain; 15 Icahn School of Medicine at Mount Sinai, One Gustave L Levy Place, P.O. Box 1030, New York, NY 10029 USA

**Keywords:** Mitral valve prolapse, Cardiovascular magnetic resonance, Mitral regurgitation, Myocardial strain

## Abstract

**Introduction:**

Mitral valve (MV) prolapse (MVP) is a primary valvular abnormality. We hypothesized that additionally there are concomitant abnormalities of the left ventricle (LV) and MV apparatus in this entity even in the absence of significant mitral regurgitation (MR).

**Objective:**

To characterize MV and LV anatomic and functional features in MVP with preserved LV ejection fraction, with and without significant MR, using cardiovascular magnetic resonance (CMR).

**Methods:**

Consecutive MVP patients (n = 80, mean 52 years, 37% males) with preserved LV ejection fraction, and 44 controls (46 years, 52% males) by CMR were included, as well as 13 additional patients with “borderline” MVP. From cine images we quantified LV volumes, MV and LV anatomic measurements (including angle between diastolic and systolic annular planes, annular displacement, and basal inferolateral hypertrophy) and, using feature tracking, longitudinal and circumferential peak systolic strains.

**Results:**

Significant MR was found in 46 (56%) MVP patients. Compared with controls, MVP patients had LV enlargement, basal inferolateral hypertrophy, higher posterior annular excursion, and reduced shortening of the papillary muscles. LV basal strains were significantly increased, particularly in several basal segments. These differences remained significant in patients without significant MR, and many persisted in “borderline” MVP.

**Conclusions:**

In patients with MVP and preserved LV ejection fraction there is LV dilatation, basal inferolateral hypertrophy, exaggerated posterior annular displacement and increased basal deformation, even in the absence of significant MR or overt MVP. These findings suggest that MVP is a disease not only of the MV but also of the adjacent myocardium.

**Supplementary Information:**

The online version contains supplementary material available at 10.1186/s12968-021-00800-w.

## Introduction

Mitral valve (MV) prolapse (MVP) is the most common cause of primary mitral regurgitation (MR) in developed countries [1]. MVP is defined as single or bileaflet prolapse, with or without leaflet thickening [2]. While MVP is primarily a disorder of the MV apparatus, few studies have suggested concomitant abnormalities in the left ventricle (LV). Increased LV diameters were described in the Framingham Study in association with MVP [3]. In addition, qualitatively abnormal LV motion patterns have been reported using angiocardiography [4] or cardiovascular magnetic resonance (CMR) [5], but quantitative measures of strain with echocardiography have yielded conflicting results [6, 7]. In addition, many of these studies did not account for the presence of significant MR as a possible mediator of observed abnormalities. In recent years CMR has emerged as an important technique for MVP characterization [2]. CMR is considered the non-invasive gold standard for LV evaluation and may allow better understanding of the interactions between the MV and LV. We hypothesized that LV morphological and functional abnormalities identified by CMR are part of the phenotypic expression of MVP even with preserved LV ejection fraction (LVEF) and in the absence of significant MR.

## Methods

### Study population

**Fig. 1:**
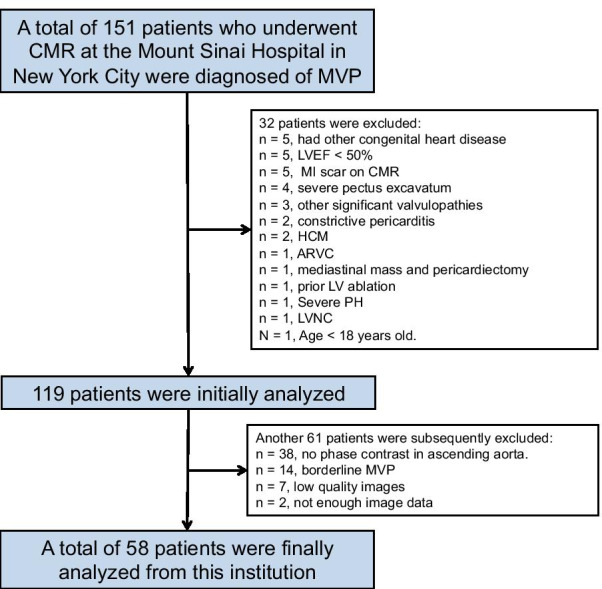
Study flow chart. Reasons for exclusion of patients screened at Mount Sinai Hospital are detailed. *ARVC*, Arrhythmogenic right ventricular cardiomyopathy; *HCM*, Hypertrophic cardiomyopathy; *LVNC*, Left ventricular non-compaction; *MI*, Myocardial infarction; *MVP*, mitral valve prolapse; *PH*, Pulmonary hypertension

Patients with CMR-based diagnosis of MVP and preserved LVEF from 2 collaborating institutions were included: the CMR program at the Mount Sinai Hospital, New York City, New York, USA, and the Centro Nacional de Investigaciones Cardiovasculares (CNIC), Madrid, Spain. Inclusion criteria were age ≥ 18 years, presence of MVP (defined as ≥ 2 mm displacement of one or both mitral leaflets into the left atrium (LA) during systole in the LV outflow tract long-axis view) [2], and LVEF ≥ 50% [8]. Exclusion criteria included: (1) conditions that could affect LV size/function and/or MV anatomy/geometry (dilated cardiomyopathy [LV dilation and systolic dysfunction in the absence of coronary artery disease or abnormal loading conditions proportionate to the degree of LV impairment [9]], prior infarct, prior mitral valve repair, additional moderate-to-severe valvular abnormalities [defined as any moderate or severe valvular stenosis or regurgitation with the exception of MR], or chest wall deformities such as pectus excavatum); (2) absence of phase contrast imaging or complete cine volumetric evaluation of the LV for quantification of MR severity; and (3) motion artifacts from respiratory motion and/or arrhythmia that precluded accurate measurements. Twenty- two patients fulfilling these characteristics were prospectively included at CNIC from October 2014 to March 2017. At Mount Sinai, a total of 151 consecutive patients with CMR based MVP diagnosis were retrospectively identified between March 2007 to January 2018. Of these, 93 were excluded (Fig. 1), resulting in 58 patients fulfilling inclusion criteria. Thus, a total of 80 patients were included. Patients were compared with a control group of 44 patients referred clinically for CMR at Mount Sinai Hospital, without evidence of structural heart disease (mild valvular regurgitation was allowed) or chest wall abnormality.

In order to evaluate the possible evolution of MVP, we additionally analyzed a separate group of patients (n = 13) with “borderline” MVP, whom we initially excluded. “Borderline” MVP was defined as morphology suggestive of prolapse but with leaflet displacement < 2 mm into the LA (a phenotype that has been linked to future MVP) [10], illustrated in Additional file 1 and 2. Of these patients, phase contrast imaging was only available in 3 and late gadolinium enhancement (LGE) imaging in 11.

Baseline demographics, cardiovascular risk factors and electrocardiographic (ECG) findings were collected from patients’ electronic health records. The respective institutional review boards approved the study. Patients enrolled at CNIC provided written informed consent, while this requirement was waived at Mount Sinai.

### CMR imaging

CMR studies were performed on 1.5 T (Magnetom Sonata or Magnetom Avanto, Siemens Healthineers, Erlangen, Germany, or Optima MR450w; General Electric Healthcare, Milwaukee, Wisconsin, USA) or 3 T (Ingenuity, Philips Healthcare, Best, The Netherlands) CMR units using dedicated phased-array surface coils as receivers. Sequences were acquired during end-expiratory breath holds with retrospective ECG or pulse gating. Balanced steady-state free-precession (bSSFP) cine images were acquired in three standard long-axis views (4-chamber, 2-chamber and LV outflow tract) in all patients. In addition, a stack of contiguous short-axis views covering the entire ventricles was available in all patients. Cine images were obtained with in-plane spatial resolution of ≤ 2 mm and temporal resolution ≤ 45 ms following current recommendations [11]. To determine the presence of significant MR, phase contrast velocity-encoded imaging was acquired orthogonal to the ascending aorta with typical velocity-encoding limit of 150 cm/s. In most patients, LGE imaging was obtained 10–15 min after the administration of 0.15–0.02 mmol/kg of gadolinium-based contrast agent.

### Image analysis

Short-axis cine and phase contrast images were analyzed using specialized software (Argus, Siemens Healthineers; Extended MR WorkSpace, Philips Healthcare; or Qmass 6.0, Medis Medical Imaging Systems, Leiden, The Netherlands). All measurements of MV geometry in the LV outflow tract view and LV strain quantification were obtained with cvi^42^ (version 5.6.3, Circle Cardiovascular Imaging, Calgary, Alberta, Canada), by the same observer.

**Fig. 2:**
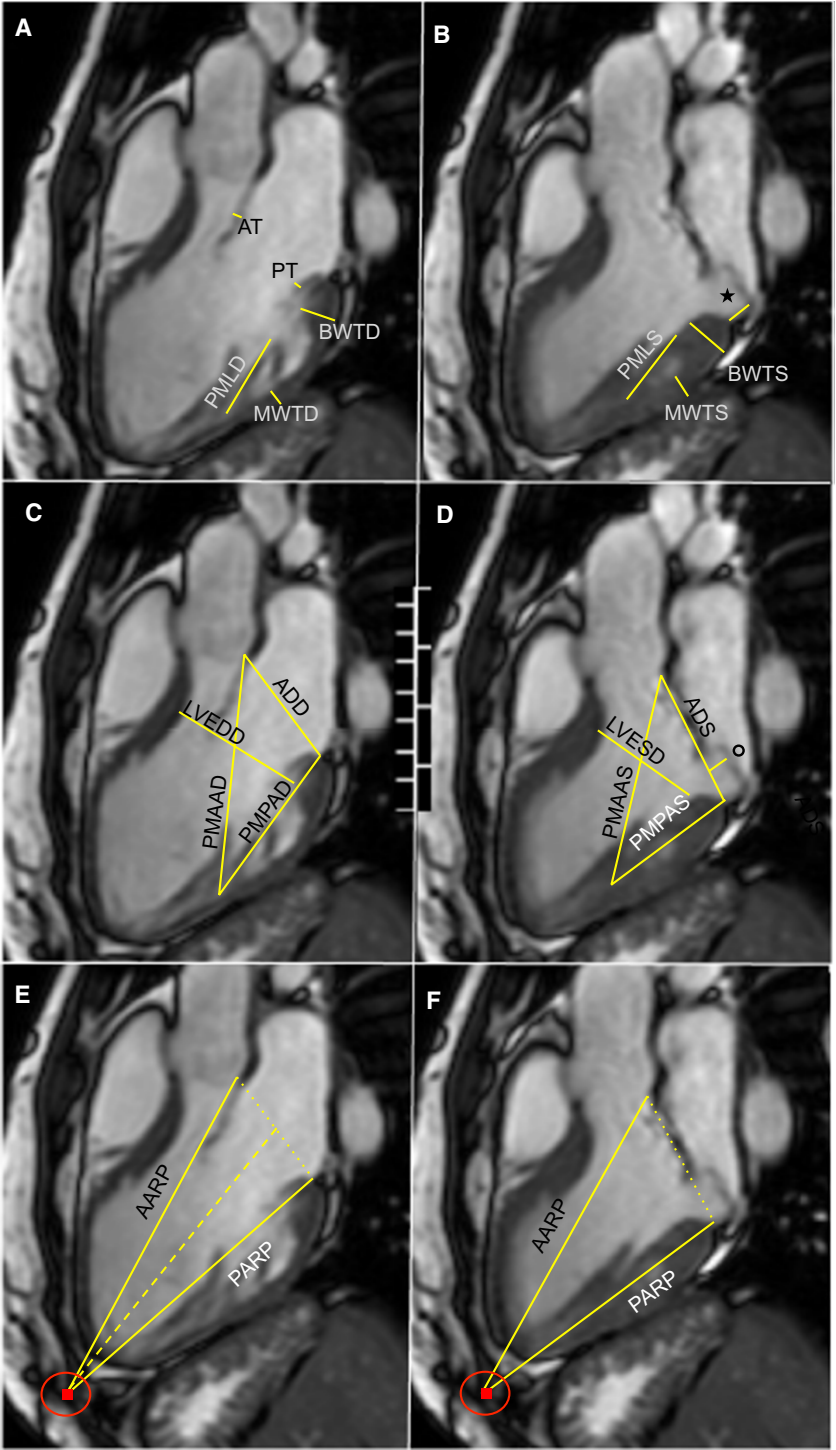
Cardiovascular magnetic resonance (CMR) balanced steady state free precession -cine left ventricular outflow tract long-axis view in a patient with posterior mitral valve prolapse. (**A** and **C**). End-diastole phase showing measurements of the mitral annulus diameter (ADD), left ventricle end-diastolic diameter (LVEDD), posteromedial papillary muscle length (PMLD), chord length from papillary muscle to the tip of the leaflet (CLD), distance from PM insertion point to the anterior and posterior mitral annulus (PMAAD and PMPAD), basal and mid inferolateral wall thickness (BWTD and MWTD), anterior and posterior leaflet thickness (AT and PT). (**B**–**D**) End-systole phase showing measurements of the mitral annulus diameter (ADS), maximum MVP (circle), left ventricle end-systolic diameter (LVESD), basal and mid inferolateral wall thickness (BWTS and MWTS), posteromedial papillary muscle length (PMLS), mitral annular disjunction (star), distance from PML insertion point to the anterior mitral annulus and to the posterior mitral annulus (PMAAS and PMPAS). Finally, end-diastole (**E**) and end-systole (**F**) phase showing the mitral annulus diameter (dotted line), and distance from the anterior (AARP) and posterior (PARP) mitral annulus to the external reference point (red dot indicates the muscle-fat interface in the chest wall)

LV mass, biventricular volumes and ejection fractions were quantified from bSSFP-cine images by manually tracing the endocardial and epicardial contours of the short axisat end-diastole and end-systole. Papillary muscles and trabeculations were considered part of the LV cavity [12]. In an LV outflow tract long-axis view we measured LV end-diastolic and end-systolic diameters as well as end-diastolic and end-systolic wall thickness of the basal inferolateral and mid inferolateral segments [13, 14] (Fig. 2). LA volume was derived by tracing end-systolic LA contours in the 4-chamber and 2-camber views and using the biplane area-length method [15]. Mitral regurgitant fraction (RF) was quantified comparing volumetric LV stroke volume to forward aortic flow in the aorta as previously described; significant MR was defined as RF > 15% [16]. The presence and location of LGE was determined visually.

Regarding MV anatomy and geometry, the following measurements were performed in the LV outflow tract long-axis view (Fig. 2):

- MVP distance: maximum displacement of the mitral leaflets beyond the annular plane during systole [2].
- Mitral annular diameters: measured from the aortomitral junction to the posterior mitral leaflet insertion point both in diastole and systole, with their difference defined as annular lengthening.
- Maximum leaflet thickness, measured in diastole.
- Length of the posteromedial papillary muscle (PM): measured in end-diastole and end-systole, with their difference defined as PM shortening.
- Distance of the tip of the posteromedial PM to the tip of the posterior mitral leaflet as a surrogate of “chordal length”, measured in end-diastole and end-systole.
- Distances from the posteromedial PM insertion point to the anterior and posterior mitral annulus. These were also measured in end-diastole and end-systole, as well as their difference.
- Distances from the anterior and posterior mitral annulus to external reference point (the point of muscle-fat interface in the chest wall on a line traversing the center of the annular plane and LV apex) were measured in end-diastole and end-systole, as well as their difference.
- Mitral annular disjunction (MAD): distance from the LA wall-mitral posterior leaflet junction to the LA wall-LV inferolateral wall junction [17], measured during diastole and systole.
- Mitral annulus angle: measured as the angle between the mitral annular planes at end-diastole and end-systole.

**Fig. 3:**
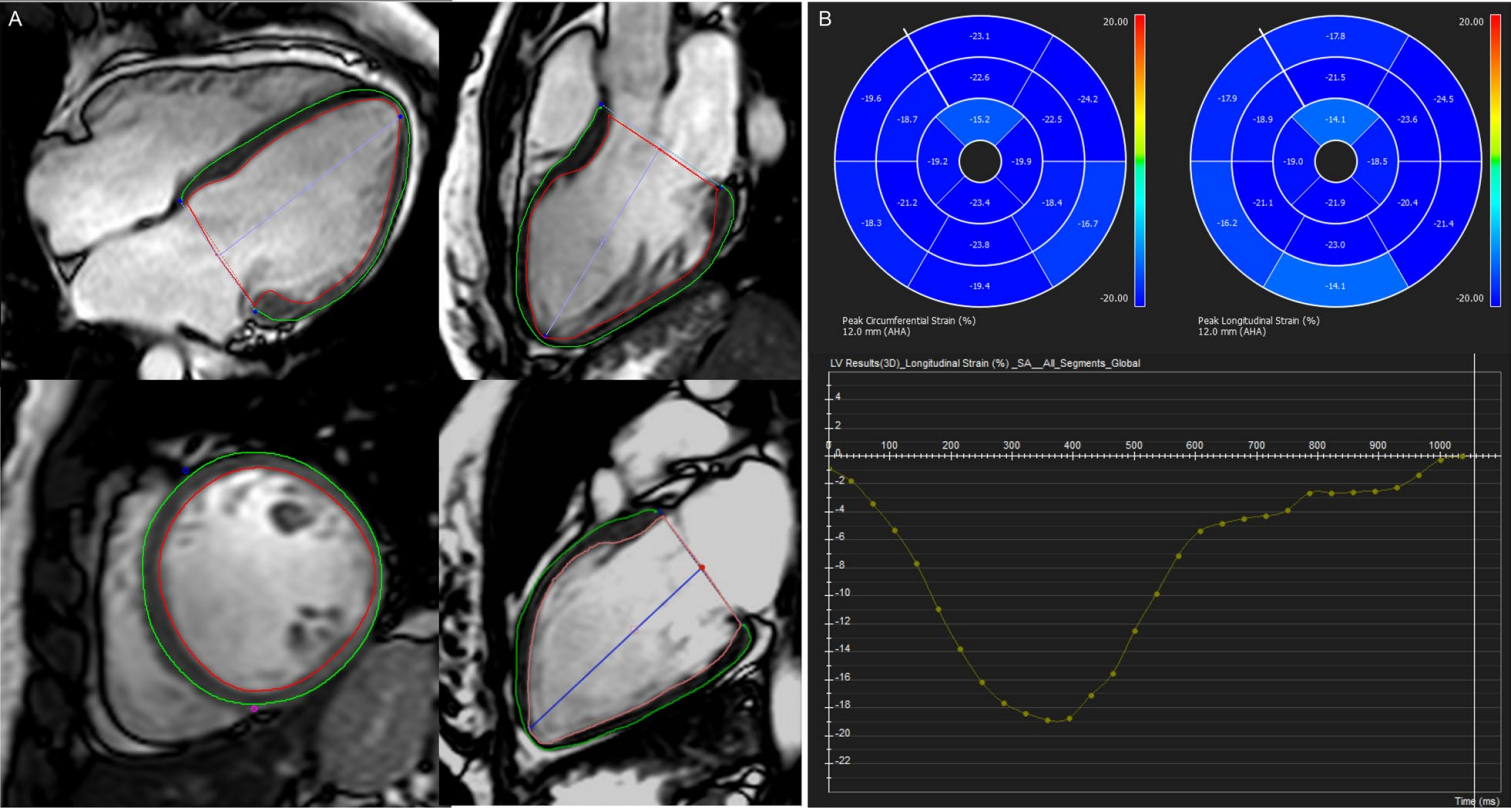
Left ventricle strain analysis by feature tracking. **A** Epicardial and endocardial borders, excluding the papillary muscles, were manually drawn in the end-diastolic phase in the 4-chamber, left ventricular (LV) outflow tract long-axis, short axis and 2-chamber views. **B** Bull´s-Eye figures representing segmental longitudinal and circumferential strain, and global peak longitudinal strain (curve) by feature tracking. *LA* left atrium

We quantified LV peak systolic longitudinal and circumferential strains using cvi^42^ feature-tracking module. Epicardial and endocardial borders, excluding the papillary muscles, were manually drawn in the end-diastolic phase in the short axis, 4-chamber, 2-chamber and LV outflow tract long-axis views, and then automatically propagated through the cardiac cycle (Fig. 3). Strains were obtained following the standard 16-segment model [18], and then averaged for the basal, mid, and apical levels as well for the entire LV.

### Statistical analysis

Categorical data are expressed as number (percentage) whereas continuous variables are presented as median [interquartile range], since most were not normally distributed according to the Shapiro–Wilk test. For comparisons between MVP patients and controls, differences for categorical variables were evaluated by the chi-square (*χ*^2^) or Fisher exact tests, and non-parametric tests such as Wilcoxon rank-sum for continuous variables as appropriate. To ensure that potential LV abnormalities were not secondary to volume overload in the presence of MR, a sub-analysis was performed comparing controls with MVP patients without significant MR. Similar comparisons were performed between controls and patients with “borderline” MVP.

Reproducibility of MV geometry and LV systolic strain measurements was assessed in 20 randomly selected patients, including 10 with MVP and 10 controls. Intraobserver variability was obtained from repeated measurements performed by the same reader at least one month apart, and interobserver variability by a second independent reader. Variability was evaluated using the intraclass correlation coefficient (ICC). The following levels of agreement were used: excellent for ICC > 0.74, good for ICC 0.6‒0.74, fair for ICC 0.4‒0.59, poor for ICC < 0.4 [19].

In all cases, a *p* value < 0.05 was considered statistically significant. The Holm-Bonferroni correction [20] was used to account for multiple testing in comparisons of segmental myocardial strain. All statistical analyses were performed with STATA (version 13.1, Stata Corporation, College Station, Texas, USA).

## Results

### Patient characteristics

The clinical characteristics of the MVP (including MVP without significant MR), control and “borderline” MVP groups are summarized in Table 1. Controls were slightly younger than the overall MVP group, and had higher body surface area and prevalence of obesity groups. Other than these measures, there were no differences regarding and sex, remaining cardiovascular risk factors or symptoms between these 2 groups.

**Table 1:** Patient characteristics

	Controls (n = 44)	MVP (all) (n = 80)	MVP (No significant MR) (n = 34)	“Borderline” (n = 13)
Age (y)	46 [38–57]	52 [40–63]*	50 [35–59]	51 [36–63]
Male	23 (52%)	37 (46%)	14 (41%)	7 (50%)
BSA (m^2^)	1.9 [1.7–2.1]	1.7 [1.6–2.0]*	1.7 [1.6–2.0]*	1.8 [1.6–1.9]*
Hypertension	18/42 (43%)	27/73 (37%)	10/31 (32%)	1/9 (11%)
Diabetes mellitus	5/42 (12%)	0/51 (0%)	0/23 (0%)	1/13 (13%)
Current or former smoker	13/42 (31%)	13/50 (26%)	6/23 (26%)	0/9 (0%)*
Dyslipidemia	12/42 (29%)	16/52 (31%)	7/23 (30%)	2/9 (22%)
Obesity	10/42 (23%)	6/80 (8%)**	3/34 (9%)*	0/13 (0%)*
Family history of MVP (%)	0/41 (0%)	5/73 (7%)	2/31 (6%)	0/9 (0%)
Dyspnea	12/41 (29%)	19/71 (27%)	7/31 (23%)	2/8 (25%)
Chest pain	9/41 (22%)	17/72 (24%)	7/32 (22%)	1/8 (13%)
Palpitations	17/41 (41%)	50/98 (51%)	18/31 (58%)	4/8 (50%)
Sinus rhythm	44/44 (100%)	78/78 (100%)	34/34 (100%)	13/13 (100%)

*BSA* body surface area. *MR* mitral regurgitation. *MVP* mitral valve prolapse

Values expressed as n (%) or medians [interquartile range]. *P Value < 0.05 versus controls. **P Value < 0.01 versus controls

### Standard CMR measurements

In the MVP cohort 46 patients had significant (at least moderate) MR by CMR. By design, no patients in the control group had significant MR. CMR measurements of cardiac chambers are summarized in Table 2. Patients with MVP, including those without significant MR, showed increased LV end-systolic and end-diastolic dimensions without significant differences in LV mass or LVEF. RV end-diastolic volume was also higher in MVP patients regardless of MR, whereas LA volumes were increased only in the overall group but not in the cohort without significant MR. LV end-diastolic basal inferolateral wall thickness was increased in patients with MVP and significant MR, and mid inferolateral wall thickness reduced in in the cohort without significant MR. However, basal inferolateral wall thickness was increased in LV end-systole and mid inferolateral wall thickness reduced in end-diastole regardless of the presence of MR, leading to increased basal-to-mid inferolateral wall thickness ratios (Table 2).

**Table 2:** CMR chamber quantification

	Controls [n = 44]	MVP (all) [n = 80]	MVP (no significant MR) [n = 34]	“Borderline” MVP [n = 13]
LVEDV (ml/m^2^)	73 [67–90]	98 [86–116]**	90 [85–101]**	83 [74–89]
LVESV (ml/m^2^)	30 [25–34]	39 [31–445**	37 [30–44]*	35 [32–37]
LVEF (%)	61 [57–65]	60 [57–66]	60 [57–64]	59 [56–62]
LV mass (g/ m^2^)	51 [42–59]	54 [46–65]	48 [43–59]	47 [41–54]
LV end-diastolic diameter (cm/ m^2^)	2.6 [2.3–2.8]	2.8 [2.5–3.2]**	2.7 [2.5–3.2]*	2.8 [2.5–2.9]*
LV end-systolic diameter (cm/ m^2^)	1.7 [1.5–2.0]	1.9 [1.7–2.2]*	1.9 [1.8–2.1]*	1.9 [1.8–2.1]
Basal inferolateral thickness in diastole (cm)	0.7 [0.6–0.9]	0.8 [0.7–1.0]*	0.7 [0.6–0.9]	0.7 [0.6–0.7]
Basal inferolateral thickness in systole (cm)	1.2 [1.1–1.4]	1.5 [1.3–1.7]**	1.5 [1.3–1.6]**	1.3 [1.2–1.4]
Mid inferolateral thickness in diastole (cm)	0.6 [0.5–0.8]	0.6 [0.5–0.7]	0.6 [0.4–0.7]*	0.4 [0.3–0.5]**
Mid inferolateral thickness in systole (cm)	1 [0.9–1.2]	1 [0.8–1.2]	1 [0.7–1.2]	0.7 [0.6–0.8]**
Basal-to-mid inferolateral end-diastolic thickness ratio	1.1 [1–1.3]	1.4 [1.1–1.7]**	1.4 [1–1.6]*	1.7 [1.3–1.7]**
Basal-to-mid inferolateral end-systolic thickness ratio	1.2 [1–1.4]	1.5 [1.2–1.7]**	1.6 [1.3–1.8]**	1.9 [1.8–2.0]**
RVEDV (ml/ m^2^)	76 [69–86]	85 [75–104]*	85 [81–101]*	74 [63–99]
RVESV (ml/m^2^)	33 [28–38]	36 [28–47]	37 [28–48]	37 [27–42]
RVEF (%)	57 [55–62]	59 [53–65]	59 [53–63]	56 [51–60]
LA volume (ml/ m^2^)	38 [30–43]	50 [40–67]**	41 [34–49]	50 [36–59]*
Mitral valve regurgitant fraction (%)	4 [0–6]	20 [11–32]*	9 [1–13]	N/A
LGE performed	44 (100%)	70 (85%)	25 (74%)	11 (85%)
Myocardial LGE	2 (5%)	23 (33%)**	8 (32%)*	3 (27%)
Intramyocardial pattern	2 (5%)	16 (23%)*	4 (16%)*	3 (27%)
Inferolateral LGE	0 (0%)	22 (32%)*	8 (33%)*	2 (18%)

Values expressed as n (%) or medians [interquartile range]

*LA* left atrium. *LGE* late gadolinium enhancement. *LV* left ventricle. *LVEDV* left ventricle end-diastolic volume. *LVEF* left ventricle ejection fraction. *LVESV* left ventricle. *N/A* not applicable. *RVEDV* right ventricular end-diastolic volume. *RVEF* right ventricular ejection fraction. RVESV: right ventricular end-systolic volume. *SAX* short axis view

*P Value < 0.05 versus controls. **P Value < 0.01 versus controls

Myocardial LGE was available in 44 controls, 70 MVP patients, and 11 patients with “borderline MVP”. There were two control subjects with focal LGE in the inferior RV insertion point, considered nonspecific. Regarding MVP patients, 23 (33%) had myocardial LGE, being intramyocardial the most common pattern and the inferolateral segment the most commonly affected (Table 2). Examples of abnormal LGE are shown in Additional files 3.

### MV apparatus geometry

MV apparatus measurements are presented in Table 3. In patients with MVP, isolated posterior leaflet involvement was the most common type (n = 45; 56%), followed by bileaflet MVP (n = 32; 40%) and isolated anterior leaflet MVP (n = 1; 1%). The median MVP distance was 0.7 cm. As expected [2], leaflet thickness and mitral annular diameters were increased in MVP. Mitral annular disjunction (MAD) was present in 42% of MVP patients versus only one (2%) patient in the control group. In addition, the MV annulus angle was increased in MVP patients regardless of MR, indicating increased excursion of the posterior annulus with respect to the mitroaortic junction. This was confirmed by increased shortening in the distance from the posterior mitral annulus to the external reference point, also in the absence of significant MR (see Table 3), a difference that was not noted for the anterior annulus.

**Table 3:** Mitral valve apparatus measurements

	Controls [n = 44]	MVP (all) [n = 80]	MVP (no significant MR) [n = 34]	“Borderline” MVP [n = 13]
MVP distance (mm)	N/A	7 [5–9]	6 [4–8]	0.9 [0.5–1.7]
Mitral leaflets thickness				
Anterior leaflet (mm)	2 [2–2]	3 [2, 3]**	3 [2, 3]**	3 [3–5]**
Posterior leaflet (mm)	1 [1, 2]	2 [2, 3]**	2 [2, 3]**	4 [3, 4]**
Mitral annulus diameter				
Diastole (cm/m^2^)	1.7 [1.5–1.9]	2.1 [1.9–2.4]**	1.9 [1.7–2.1]**	2.1 [1.9–2.4]*
Systole (cm/m^2^)	1.9 [1.7–2.1]	2.4 [2.1–2.7]**	2.0 [1.9–2.2]**	1.9 [1.6–2.1]
Lengthening (cm/m^2^)	0.2 [0–0.4]	0.2 [0.1–0.4]	0.3 [0.1–0.4]	0.4 [0.3–0.5]*
Mitral annulus disjunction				
Presence diastole, n (%)	1 (2)	14 (18)**	7 (21)**	2 (15)*
Extent diastole (cm)	0.2 [0.2–0.2]	0.3 [0.2–0.3]*	0.3 [0.2–0.3]*	0.3 [0.2–0.3]
Presence systole, n (%)	1 (2)	32 (40)**	15 (44)**	4 (31)**
Extent systole (cm)	0.2 [0.2–0.2]	0.8 [0.6–1.0]**	0.7 [0.4–1]**	0.6 [0.2–0.6]
Mitral annulus angle (º)	4 [2–6]	7 [4–10]**	7 [3–11]*	3 [2–6]
Anterior annulus to external reference point				
Diastole (cm/m^2^)	6.6 [6.2–7.2]	7.1 [6.6–7.8]*	7.2 [6.5–8.0]*	7.3 [7.2–7.7]*
Systole (cm/m^2^)	6.0 [5.6–6.7]	6.5 [5.9–7.1]*	6.5 [5.7–7.4]	6.9 [6.5–6.9]*
Shortening (cm/m^2^)	0.6 [0.5–0.8]	0.6 [0.5–0.8]	0.6 [0.5–0.8]	0.8 [0.6–0.9]
Posterior annulus to external reference point				
Diastole (cm/m^2^)	6.4 [6.0–6.8]	6.8 [6.2–7.4]*	6.8 [6.2–7.3]*	7.2 [6.9–7.3]**
Systole (cm/m^2^)	5.6 [5.3–6.2]	6.0 [5.4–6.5]	6.0 [5.3–6.5]	6.5 [6.1–6.6]*
Shortening (cm/m^2^)	0.7 [0.5–0.9]	1.0 [0.7–1.0]*	0.9 [0.7–1.0]*	0.8 [0.6–1.1]
PM to anterior annulus distance				
Diastole (cm/m^2^)	3.8 [3.7–4.1]	4.4 [4.1–4.8]**	4.4 [4.1–4.7]**	4.4 [3.9–4.4]*
Systole (cm/m^2^)	3.4 [3.2–3.8]	3.6 [3.3–4.0]	3.5 [3.3–3.9]	3.6 [3.4–3.9]
Shortening (cm/m^2^)	0.5 [0.3–0.6]	0.8 [0.6–1.0]**	0.8 [0.6–0.9]**	0.7 [0.4–0.8]
PM to posterior annulus distance				
Diastole (cm/m^2^)	3.1 [2.9–3.3]	3.4 [3.1–3.8]**	3.3 [3.1–3.6]*	3.4 [2.8–3.5]
Systole (cm/m^2^)	2.6 [2.4–2.8]	2.7 [2.4–2.9]	2.6 [2.4–2.8]	2.8 [2.5–3.1]
Shortening (cm/m^2^)	0.5 [0.3–0.6]	0.8 [0.6–1.0]**	0.7 [0.5–0.9]**	0.4 [0.4–0.8]
PM length				
Diastole (cm/m^2^)	1.6 [1.4–1.7]	1.7 [1.4–1.9]	1.7 [1.4–1.8]	1.6 [1.4–1.9]
Systole (cm/m^2^)	1.2 [1.0–1.4]	1.5 [1.2–1.7]**	1.4 [1.2–1.6]*	1.3 [1.2–1.5]
Shortening (cm/m^2^)	0.4 [0.2–0.5]	0.2 [0.1–0.4]*	0.3 [0.1–0.4]*	0.3 [0.3–0.4]
Distance from papillary muscle tip to leaflet				
Diastole (cm/m^2^)	1.1 [0.9–1.2]	1.1 [1.0–1.3]	1.1 [0.9–1.2]	1.1 [1.1–1.3]*
Systole (cm/m^2^)	1.1 [0.9–1.2]	1.2 [1.1–1.4]*	1.2 [1.0–1.4]	1.2 [1.1–1.4]*

Values expressed as medians [interquartile range]. *P Value < 0.05 versus controls. **P Value < 0.01 versus controls. *MR* mitral regurgitation, *MVP* mitral valve prolapse; *N/A* not applicable, *PM* papillary muscle

The end-diastolic distance PM to both anterior and posterior mitral annulus was longer in MVP patients even in the absence of significant MR. However, there were no significant end-systolic differences, leading to increased shortening in the PM-annulus distances in MVP. Conversely, PM length was preserved in end-diastole but increased in end-systole in MVP patients, resulting in reduced PM shortening. Systolic distance between the tip of the PM and the posterior mitral leaflet (“chordal length”) was increased in the overall MVP group but not in those without significant MR, with no systolic differences in either group.

### LV myocardial strain

**Fig. 4:**
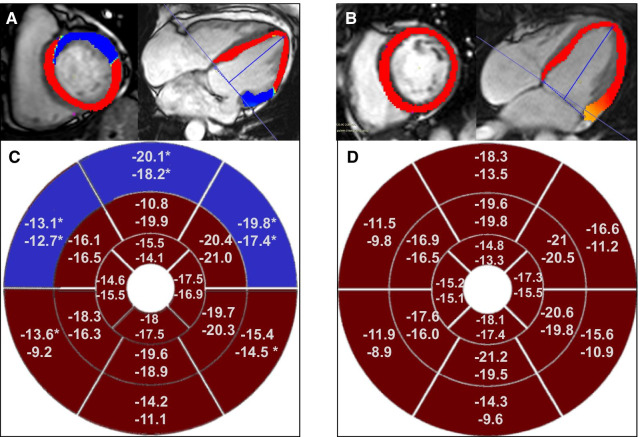
**C**ircumferential and longitudinal peak systolic strain analysis, representing the differences between MVP (**A**) and controls (**B**). Bull´s-Eye figures representing American Heart Association LV segmentation, with longitudinal and circumferential peak systolic strain. Values are shown for MVP cases (**C**) and controls (**D**). *LV* Left ventricle. *MVP* Mitral valve prolapse. *P Value < 0.05 versus controls

Global strain values, as well as those segmental strains showing significant differences between controls and MVP patients (including those without significant MR) are shown in Table 4. There were no differences in the global longitudinal strain or global circumferential strain measurements; however, the MVP group had increased basal longitudinal strain values regardless of the presence of MR, and increased basal circumferential strain in the overall cohort. When evaluating segmental strains, differences were significant in the overall cohort for longitudinal strain in the basal anteroseptal, anterior, anterolateral, and inferolateral segments, in the overall cohort for circumferential strain in the basal anterior and anterolateral segments, and in the group without MR for circumferential strain in the basal anterolateral segment. (Table 4, Fig. 4).

**Table 4:** Left ventricle peak systolic longitudinal and circumferential strain

LV peak systolic longitudinal strain (%)				
	Controls [n = 44]	MVP (all) [n = 78]	MVP (no significant MR) [n = 34]	“Borderline” MVP [n = 13]
Global	− 13.9 [− 15.5/− 11.6]	− 14.8 [− 16.9/− 13.1]	− 14.3 [− 16.0/− 13.1]	− 15 [− 16.6/− 13.5]
Basal	− 10.4 [− 12/− 8.5]	− 13.7 [− 17.5/− 11.3]**	− 12.9 [− 16.2/− 9.5]*	− 13.5 [− 14.8/− 11.9]**
Basal anterior	− 13.5 [− 16.3/− 10.6]	− 18.2 [− 20.5/− 14.6]°	− 16.5 [− 19.5/− 10.8]	− 15.1 [− 17.9/− 13.5]
Basal anterolateral	− 11.5 [− 14.9/− 8.0]	− 17.4 [− 20.9/− 11.7]°	− 16.5 [− 21.0/− 9.4]	− 15.9 [− 17.4/− 12.2]°
Basal inferolateral	− 10.9 [− 13.4/− 9.1]	− 14.5 [− 18.1/− 11.8]°	− 13.2 [− 17.2/ − 11.7]	− 13.7 [− 18.2/− 8.9]
Basal anteroseptal	− 9.8 [− 11.8/− 5.2]	− 12.7 [− 15.5/− 8.8]°	− 11.4 [− 15.1/− 7.3]	− 9.9 [− 14.4 /—9.2]

LV peak systolic circumferential (strain %)				
	Controls (n = 43)	MVP (all) (n = 78)	MVP (no significant MR) (n = 34)	“Borderline” MVP [n = 13]
Global	− 16.4 [− 17.6/− 13.8]	− 16.23 [− 18.5/− 14.8]	− 15.8 [− 17.1/− 14.8]	− 16.6 [− 17.3/− 14.7]
Basal	− 14.6 [− 16/− 12.1]	− 16.2 [− 17.6/− 14.1]*	− 15.3 [− 16.8/− 14.2]	− 16.6 [− 18.1/− 14.7]*
Basal anterior	− 18.3 [− 19.6/− 15.0]	− 20.1 [− 22.4/ − 17.8]°	− 19.6 [− 22.4/− 17.6]	− 17.9 [− 20/− 16.3]
Basal anterolateral	− 16.6 [− 19.6/− 13.4]	− 19.8 [− 21.9/− 17.4]°	− 19.9 [− 22.5/− 17.6]°	− 18.9 [− 22.1/− 17.4]

Values expressed as medians [interquartile range]. *P Value < 0.05 versus controls. **P Value < 0.01 versus controls. °*P* Value < 0.05 versus controls after Holm-Bonferroni correction. *LV* left ventricle, *MR* mitral regurgitation; *MVP* mitral valve prolapse. Other regional strains are shown in the Additional files 4 and 5

There were no other consistent significant differences regarding segmental peak systolic strains (see Fig. 4, and Additional files 4 and 5).

In simple linear regression analysis, there was not significant correlation between global or segmental longitudinal peak systolic strain and MVP severity (*p* > 0.05, for all segments). Regarding MV leaflets thickness; there was an increase of -13% and -15% in peak systolic longitudinal strain in basal segments per every increase of 1 mm in mitral valve anterior and posterior leaflet thickness, respectively (*p* < 0.05).

We also compared strain values between those with isolated posterior versus bileaflet MVP to evaluate if there were differences in myocardial deformation between these 2 groups. As demonstrated in Additional file 6, we did not find any significant differences.

### Patients with “borderline” MVP

Patients with “borderline” MVP shared some of the differences of MVP patients in comparison with controls (Tables 1, 2, 3 and 4). Regarding clinical characteristics, they also had lower body surface area and less obesity. On cine imaging they displayed some degree of LV and LA enlargement, increases in LV end-diastolic and end-systolic basal-to-mid inferolateral thickness ratio, and higher prevalence of LGE than controls (although the latter not-statistically significance probably due to small numbers) with a pattern and distribution that resembled that of MVP. Regarding MV anatomy and geometry, patients with “borderline” MVP also demonstrated thicker leaflets, larger annular diameters, more frequent MAD, increased distance from the posterior and anterior annulus to external reference point as well as increased shortening in the case of the posterior annulus, increased diastolic distance of the anterior annulus to the PM, larger systolic PM length, and increased distance from the PM tip to the posterior leaflet tip (“chordal length”). In addition, they demonstrated increased longitudinal strains in the basal anterolateral segment, and increased circumferential strain in the base when considered globally.

### Interobsever and intraobserver reproducibility

In both inter- and intra-observer analyses, the reproducibility of strain and MV measurements was good to excellent on average. For longitudinal peak systolic strain measurements, the median inter-observer ICC was 0.74 [0.56–0.85] and intra-observer ICC was 0.74 [0.56–0.79]. Regarding circumferential peak systolic strain, the corresponding ICCs were 0.83 [0.72–0.90] and 0.77 [0.66–0.85], respectively. Finally, of MV measurements demonstrated median inter-observer ICC of 0.79 [0.64–0.94] and intra-observer ICC of 0.77 [0.66–0.88].

## Discussion

**Fig. 5:**
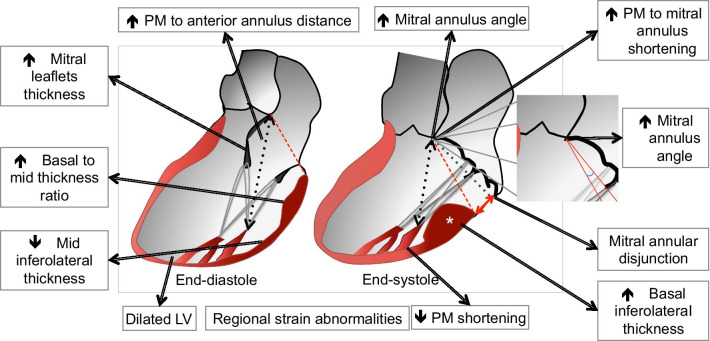
Anatomic and functional features of mitral valve. Schematic diagram of the LV outflow tract long-axis view during diastole and systole of bileaflet MVP, showing the main functional and anatomic characteristics during the cardiac cycle. The red double arrow line and white asterisk indicate mitral annular disjunction and bulging of the basal inferolateral segment, respectively. The black double arrow dotted line indicates the PM to anterior annulus distance. The mitral plane both in end diastole and end-systole is indicated y the red lie. These two planes have been superimposed in the insert illustrating the mitral annulus angle (in blue) as the angle between the two. *LV* left ventricle. *PM* papillary muscle

MVP is considered a primary disorder of the MV, with thickening and hypermobility of the leaflets. Although the importance of leaflet abnormalities cannot be downplayed, our findings suggest MVP is also associated with abnormalities and hypermobility of the annulus and adjacent myocardium (Fig. 5). This is supported by (1) increased ventricular dimensions; (2) abnormal myocardial phenotype with basal inferolateral hypertrophy, mid inferolateral thinning, and thus higher basal-to-mid inferolateral wall thickness ratio; (3) increased LV basal strains, particularly of the anterior, anterolateral and inferolateral segments; (4) increased posterior annular excursion (as reflected by a higher mitral annulus angle and shortening with respect to an external reference point); and (5) presence of MAD. Importantly, these differences were significant regardless of the presence of significant MR (and cannot be attributed to LV volume overload) and many were observed in patients with borderline MVP, suggesting that they are not secondary to full-blown leaflet prolapse.

In the Framingham Heart Study [3], 83 participants with MVP had larger LV and LA echocardiographic size than age- and sex-matched controls, although presence or severity of MR were not accounted for. Similar findings were reported more recently in 101 MVP patients evaluated with CMR [6]. In a smaller (n = 20) echocardiographic study [21], patients with MVP also had increased LV dimensions even in the absence of MR. In the present study of 80 patients with MVP as the only significant cardiac abnormality and 44 controls without structural heart disease, we also found higher LV volumes regardless of MR presence, although the LA was enlarged only in the presence of significant MR. Interestingly, a similar phenotype of a (typically mild) dilated cardiomyopathy has been observed in Marfan patients, who not uncommonly also present with MVP [22]. In addition, we noted increases in RV volumes in the MVP population even without significant MR which, to the best of our knowledge, has not been described before. Although the reasons for this are unclear, these findings may support our hypothesis that MVP represents a combination of abnormalities of not only the mitral apparatus but also cardiac chambers.

Hypertrophy confined to the basal inferolateral LV wall was initially described with echocardiography and thought to represent increased LV mass or, subsequently, a form of hypertrophic cardiomyopathy [23]. More recently and using CMR in 63 MVP patients and 20 controls, Zia et al. described LV basal hypertrophy and increased basal-to-mid thickness ratio in all LV walls, particularly anterolateral and inferolateral [14]. The severity of basal inferolateral hypertrophy correlated with the degree of annular excursion (similar to the correlation we found with MV annular angle), so focal hypertrophy was hypothesized to be adaptive to increased regional myocardial function. In our patients we also noted localized basal inferolateral hypertrophy, which, together with mid inferolateral thinning, led to increased basal-to-mid inferolateral thickness ratio. While these differences were more evident in systole, in favor of increased contractile function of the basal inferolateral segment, we also observed reduced end-diastolic thickness of the mid inferolateral segment, which suggests that phenotypic myocardial abnormalities in MVP extend beyond the perivalvular myocardium. In agreement with others [24, 25], we also observed the presence of LGE in MVP, particularly with a non-ischemic distribution and in the basal inferolateral segments, and a finding that has been linked to ventricular arrhythmias. The reasons for this are unclear but may be related to hypercontractility in this region, increased mechanical stress and, eventually localized hypertrophy.

The differences in regional LV function were confirmed by consistent increases in longitudinal and/or circumferential peak systolic strain in the basal anterior, anterolateral and inferolateral segments, and reductions in deformation in the mid inferior segment regardless of the presence of significant MR. However, we did not find differences in global strains, as opposed to other series of patients with predominantly moderate to severe MR [6, 26]. To the best of our knowledge, our study is the first study to characterize regional LV deformation in MVP patients with and without significant MR using CMR. Several studies using speckle-tracking echocardiography for evaluation of regional strain in MVP have yielded conflicting results. Fukuda et al. [27] reported reduced basal strains in association with annular dilatation in a cohort of 130 MVP patients, although they tended to have significant MR and LV dilatation. In patients with MVP and preserved LVEF, Zito et al. noted increased global circumferential strain and LV twist in the presence of moderate MR, with trends for reductions when MR was severe [28]. Lee et al. [6] found no differences in basal circumferential strain in MVP with preserved LVEF when compared to controls. Similarly, no differences in peak global or segmental longitudinal strain were noted in another study of MVP patients also with preserved LVEF, although global strain rates were increased [29].

SanFilippo et al. [30] and later Lee et al. [31] described increased shortening of the PM tip-annulus distance related to decreases only in systole. Because the annular plane did not demonstrate increased excursion with respect to an external reference point, the authors concluded that this was due to traction of the PM towards the MV rather than annular hypermobility towards the apex. Indeed, we found elongation of the PM in systole likely related to increased traction, as well reduced PM longitudinal shortening. However, we also noted as well as increased diastolic PM-annular distance, which was seen consistently and regardless of MR presence. Thus, in our study, the increased shortening of the PM-annulus distance during the cardiac cycle was due to diastolic increases rather than reductions in systole, as also noted by others [32]. As mitral chordae may be elongated in MVP, we measured the distance of the tip of the posteromedial PM to the tip of the posterior leaflet. We indeed found an increase in this distance during systole, suggesting that increased traction and mechanical stress during this period, with no differences during diastole where the chordae would not be fully stretched. Therefore, chordal elongation would not explain the increased distance from the PM base to the mitral annuls that we observed only in diastole. The augmented basal lateral strain discussed above was accompanied by increased longitudinal excursion of the posterior annulus (Fig. 2). In the normal MV the posterior annulus has larger longitudinal translation than the anterior annulus, which is tethered to the aortic root [33], but this difference is significantly accentuated in MVP. These findings support a component of relative hypermobility of the posterior annulus portion with “traction” of the PM from the more “fixed” anterior annulus. The differences between the study by SanFilippo and ours may be explained by different reasons. First, they measured the distance from the tip of the PM rather than its insertion as we did; thus, the influence of PM elongation or shortening was not accounted for. Second, the distance was measured to the center of the annular plane, which would prevent identifying differential behavior of its anterior and posterior components. Third, their study included 13 MVP patients and 18 controls studied with 2D echocardiography, whereas we enrolled 80 MVP patients and 44 controls evaluated with CMR.

**Fig. 6:**
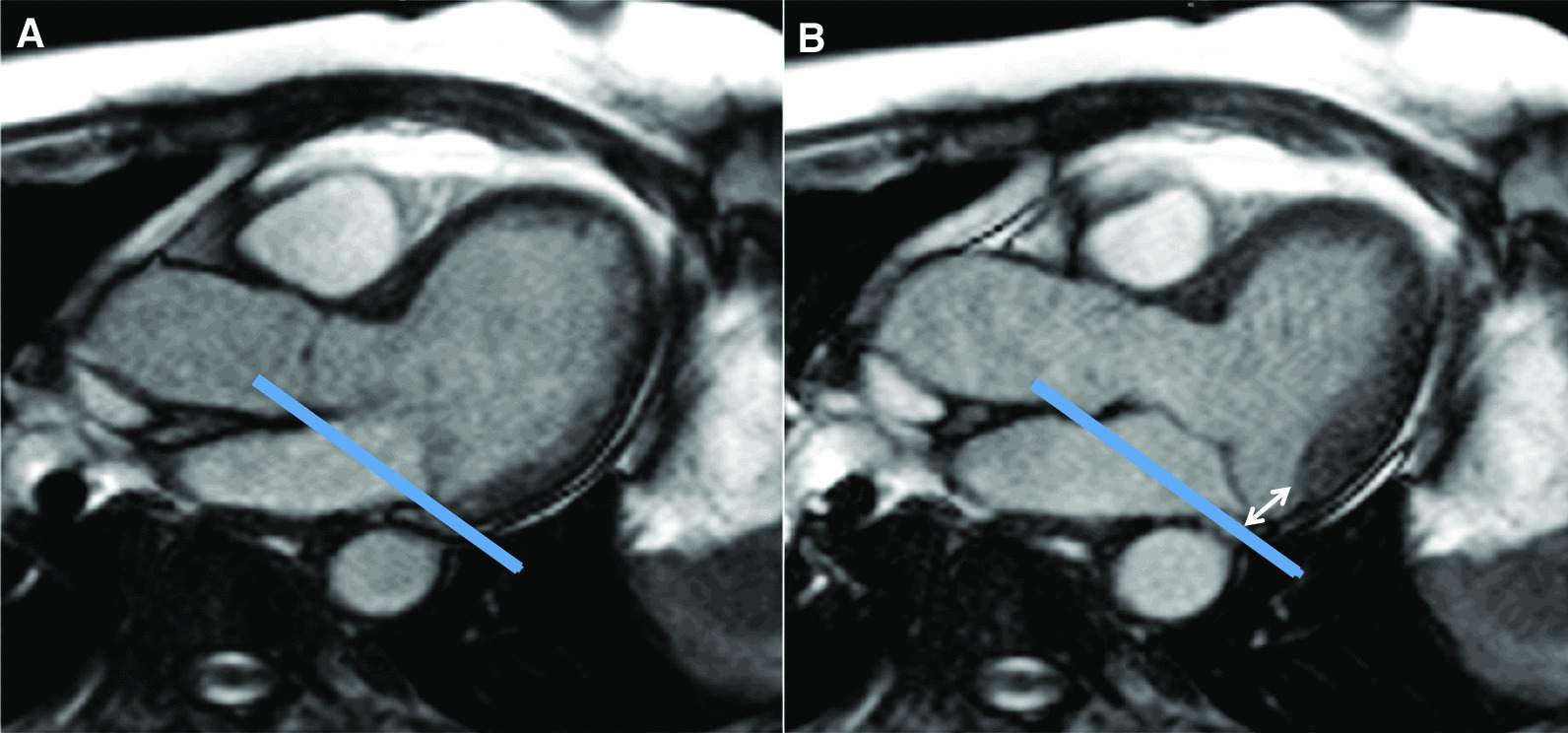
Relation of mitral leaflet and LV basal inferolateral wall motion to early-systolic mitral annular plane. **A** End-diastole still frame in the 3-chamber view, showing how the posterior mitral leaflet and basal inferolateral segment overlap the annular plane (blue line). **B** Early end-systolic still frame where the annular plane has been kept in the same anatomical position as in (**A**). While the basal inferolateral wall has moved apically from the annular plane (white arrows), the leaflet remains in the same position, suggesting inadequate “tracking” of the posterior mitral annulus by the leaflet (decoupling). The complete cine is available in the Additional file 4

A dissociation of annular and LV contraction suggesting ventricular annular decoupling has been described in MVP and related to MAD [6, 34] which in turn has been associated with more severe MVP [6], basal inferolateral abnormal motion (“curling”), regional hypertrophy [5] and MR [35]. The concept of MAD was introduced by Bharati et al. [36] in 1981, with subsequent pathology studies confirming this abnormality both in hearts with and without MVP [17]. The authors of the latter study hypothesized that MAD enables systolic annular expansion leading to changes in posterior leaflet geometry that favor prolapse and eventually secondary leaflet thickening. Our findings are consistent with this hypothesis. In fact, visually it often appears that MVP is actually consequence of excessive apical annular displacement rather than atrial leaflet displacement (Fig. 6 and Additional file 7). We hypothesize that annular and basal LV hypermobility (particularly of the posterior components) may be a primary component of the MVP complex, as opposed to secondary to leaflet prolapse. Posterior hypermobility is supported by increased of LV basal strains, of mitral annulus angles and of the shortening of the distance from the annulus to an external reference point, even in the absence of frank MVP (or significant MR). This may reflect LV-annular decoupling (which can manifest anatomically as MAD) and reduced “anchorage” of the LV basal myocardium to the MV annulus. This disruption of the “ventriculo-mitral unit” may lead to excessive systolic LV motion [5] towards the apex, insufficiently opposed by the mitral closing forces in the opposite direction and, eventually, PM elongation, increases in the diastolic PM-annulus distance, and compensatory regional hypertrophy. These findings may partly explain why annular stabilization through prosthetic annuloplasty or other techniques is an important component in the MVP surgical repair [37].

### Study limitations

Although the patients were recruited prospectively in CNIC, those at Mount Sinai were identified retrospectively, so not all clinical data were available in every patient, and some degree of referral bias is to be expected. Because of the design of the study, LGE imaging was not available in all patients. In addition, the presence of interstitial fibrosis with T1 mapping has been reported in MVP [38] and associated as well with ventricular arrhythmias [38, 39], but could not be studied in our cohort because most patients were scanned before this option was available for clinical use at Mount Sinai. Nonetheless, the role of replacement and interstitial fibrosis in MVP is beyond the scope of this work. When measuring MV geometry/anatomy and strain, the observer was blinded to the quantitative analysis of LV volumes or aortic flow. However, blinding to the presence of MVP was not possible since this can be detected in the cine images employed for the analyses. We observed mild RV dilatation in MVP. Because patients with known or CMR-based significant valvular abnormality other than MR were excluded, and echocardiographic data that was available in 67% of controls and 54% of MVP patients demonstrated right ventricular systolic pressures of 28 [21–31] mmHg and 26 [19–30] mmHg, respectively (data not shown), we speculate that tricuspid regurgitation or pulmonary hypertension do not explain these findings. However, this possibility was not tested formally and cannot be completely excluded. While the findings in patients with “borderline” MVP suggest that LV morphological and functional abnormalities may precede, and thus are not secondary to, full leaflet prolapse, this was a cross-sectional study, longitudinal follow up is needed to confirm these observations and given the small number of patients, findings in this subgroup should be considered exploratory and hypothesis-generating. CMR cannot replace the easy applicability of the echo as bedside examination; however, the higher resolution of the CMR images combined with the multiplanar dynamic series can be an excellent "basic science" tool in this delicate and fundamental chapter of cardiac mechanics.

## Conclusions

MVP is a complex entity, with abnormalities not limited to the valve apparatus, but of the whole “ventriculo-mitral unit”. Morphological and functional abnormalities of the posterior annulus and adjacent basal myocardium (basal inferolateral hypertrophy, mid inferolateral thinning, MAD, hypermobility, increased strain) appear to be a central component of MVP and may have a role in its pathogenesis.

## Supplementary Information

**Additional file 1. **Borderline mitral valve prolapse. Movies showing examples of patients with mitral leaflet morphology suggestive of prolapse but posterior displacement into the let atrium < 2 mm.

**Additional file 2. **Borderline mitral valve prolapse. Movies showing examples of patients with mitral leaflet morphology suggestive of prolapse but posterior displacement into the let atrium < 2 mm.

**Additional file 3. **Late gadolinium enhancement in patients with mitral valve prolapse**. **Examples of 4 patients with MVP and typical examples of intramyocardial LGE (asterisks) in the basal inferolateral (**A** and **B**), basal inferior (**C**) and basal anterolateral (**D**) segments. An example of papillary muscle LGE is also shown in **C** (arrow).

**Additional file 4. **Left ventricle peak systolic longitudinal strain. Table including individual LV segments peak systolic longitudinal strain measurements.

**Additional file 5. **Left ventricle peak systolic circumferential strain. Table including individual LV segments peak systolic circumferential strain measurements.

**Additional file 6. **Geometric and myocardial strain comparison between mitral valve prolapse patients with phase contrast in the ascending aorta and controls**.** Table showing the comparison between controls, MVP patients, and MVP without significant MR corroborated by phase contrast.

**Additional file 7. **Excessive apical annular displacement in MVP. Movie showing excessive apical annular displacement (rather than atrial leaflet displacement) in a patient with MVP.

## Data Availability

The datasets used and/or analyzed during the current study are available from the corresponding author on reasonable request.

## Abbreviations

BSA: Body surface area
bSSFP: Balanced steady state free precession
CMR: Cardiovascular magnetic resonance
ECG: Electrocardiogram
ICC: Intraclass correlation coefficient
LA: Left atrium/left atrial
LGE: Late gadolinium enhancement
LV: Left ventricle/left ventricular
LVEDD: Left ventricular end-diastolic dimension
LVEDV: Left ventricular end-diastolc volume
LVEF: Left ventricle ejection fraction
LVESV: Left ventricular end-systolic volume
MAD: Mitral annular disjunction
MR: Mitral regurgitation
MV: Mitral valve
MVP: Mitral valve prolapse
PM: Papillary muscle
RF: Regurgitant fraction
RV: Right ventricle/right ventricular
RVEDV: Right ventricular end-diastolic volume
RVEF: Right ventricular ejection fraction
RVESV: Right ventricular end-systolic volume
