# Supplementary figures and images for: Mitral valve prolapse morphofunctional features by cardiovascular magnetic resonance: more than just a valvular disease
Source: J Cardiovasc Magn Reson. 2021 Oct 11;23:107. doi: 10.1186/s12968-021-00800-w (PMC8504058; doi:10.1186/s12968-021-00800-w)

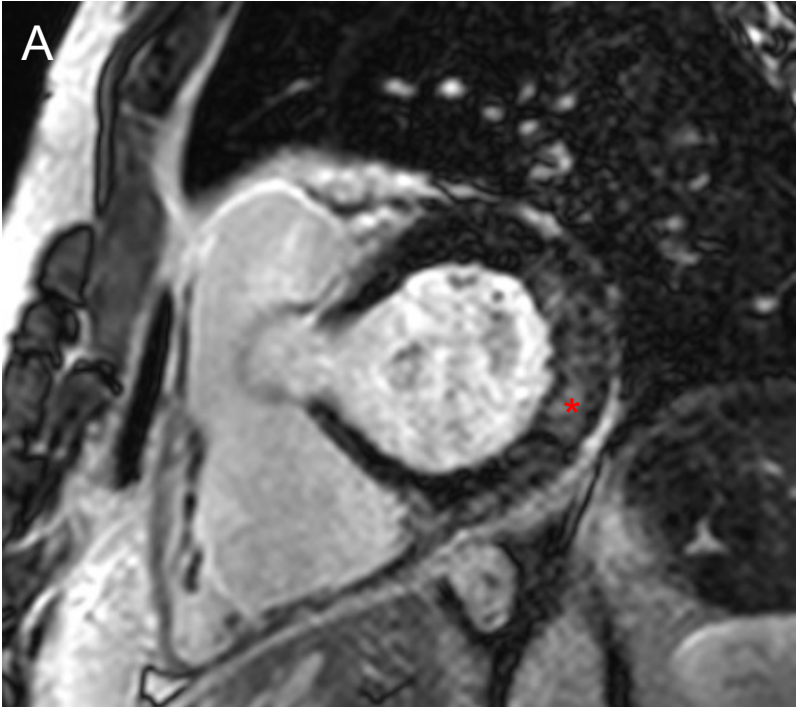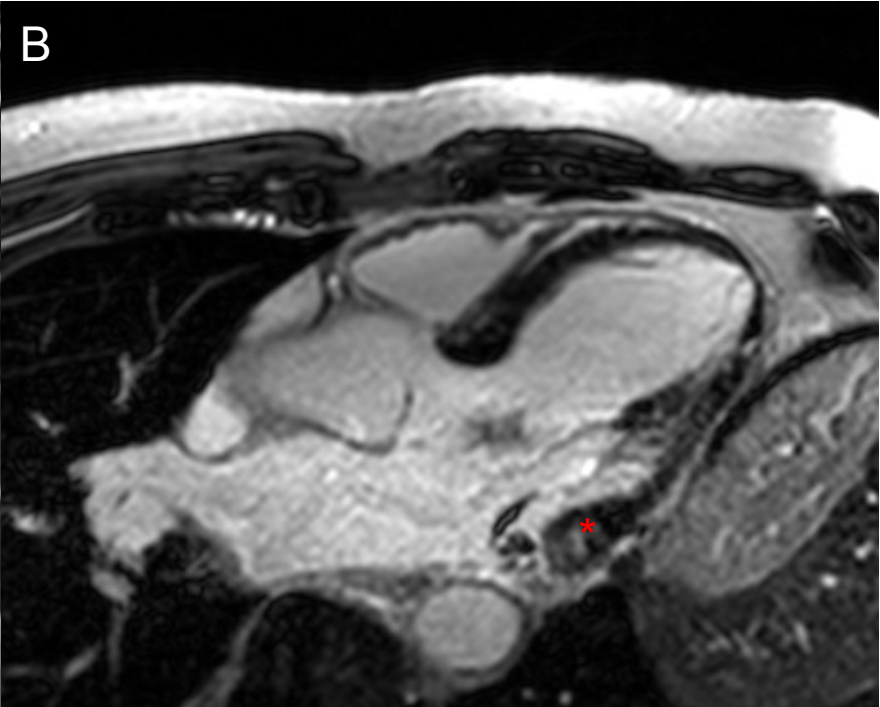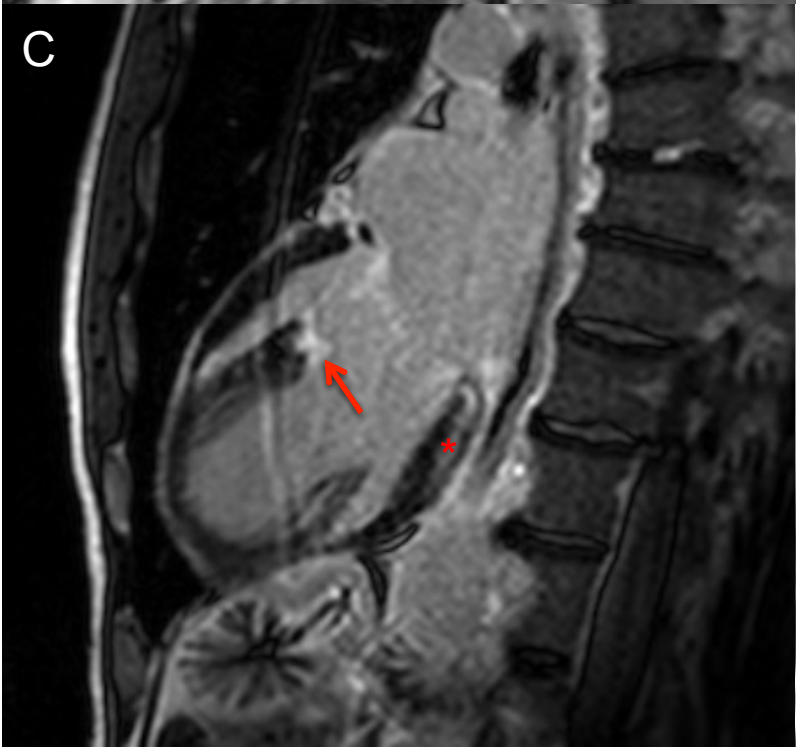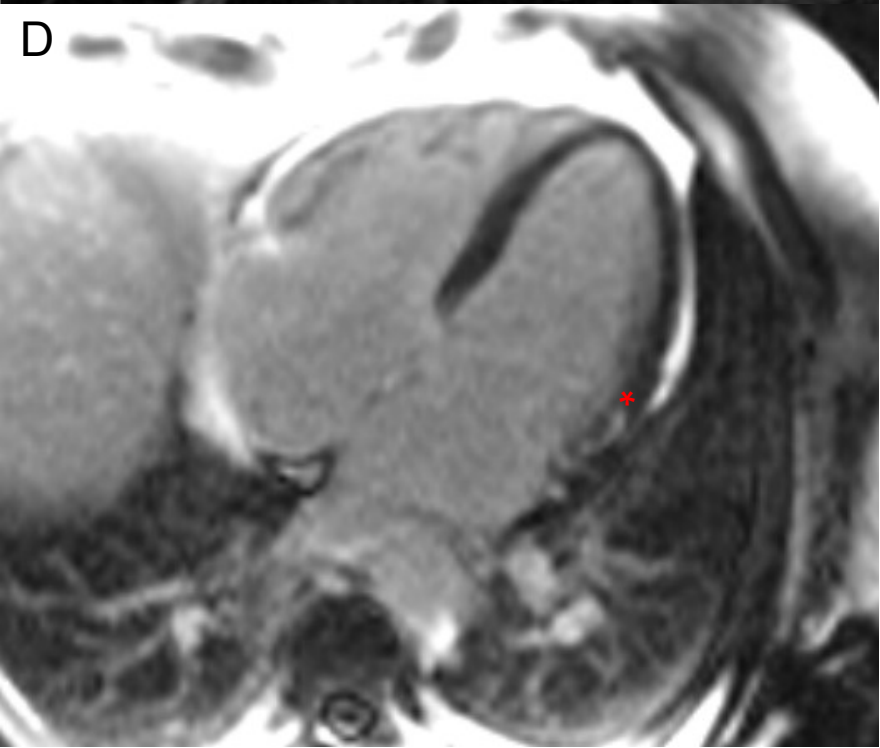

Supplement: Supplementary file 3 — Additional file 3. Late gadolinium enhancement in patients with mitral valve prolapse. Examples of 4 patients with MVP and typical examples of intramyocardial LGE (asterisks) in the basal inferolateral (A and B), basal inferior (C) and basal anterolateral (D) segments. An example of papillary muscle LGE is also shown in C (arrow). [file 12968_2021_800_MOESM3_ESM.pdf]
