# Supplementary material for: Mitral valve prolapse morphofunctional features by cardiovascular magnetic resonance: more than just a valvular disease
Source: J Cardiovasc Magn Reson. 2021 Oct 11;23:107. doi: 10.1186/s12968-021-00800-w (PMC8504058; doi:10.1186/s12968-021-00800-w)
Supplement: Supplementary file 4 — Additional file 4. Left ventricle peak systolic longitudinal strain. Table including individual LV segments peak systolic longitudinal strain measurements. [file 12968_2021_800_MOESM4_ESM.docx]

**Additional file 4. Left ventricle peak systolic longitudinal strain.**

| LV peak systolic longitudinal strain (%) | | | | |
| --- | --- | --- | --- | --- |
|  | Controls  (n=43) | MVP (all)  (n=78) | MVP (no significant MR)  (n=34) | “Borderline” MVP  [n=11] |
| Global | -13.9 [-15.5 / -11.6] | -14.8 [-16.9 / -13.1] | -14.3 [-16.0 / -13.1] | -15 [-16.6 / -13.5] |
| Basal | -10.4 [-12 / -8.5] | -13.7 [-17.5 / -11.3]** | -12.9 [-16.2 / -9.5]* | -13.5 [-14.8 / -11.9]** |
| Mid | -17.8 [-20 / -15.9] | -18.1 [-20.6 / -15.9] | -17.6 [-19.4 / -15.9] | -17.0 [-18.6 / -15.0] |
| Apical | -15.1 [-17.2 / -12.0] | -15.8 [-18.1 / -13.6] | -15.8 [-18.1 / -13.8] | -16.0 [-17.8 / -15.2] |
| Basal anterior | -13.5 [-16.3 / -10.6] | -18.2 [- 20.5 / -14.6]° | -16.5 [-19.5 / -10.8] | -15.1 [-17.9 / -13.5] |
| Basal anterolateral | -11.2 [-14.9 / -8.9] | -17.4 [-20.9 /-11.7]° | -16.5 [-21.0 / -9.4] | -15.9 [-17.4 / -12.2]° |
| Basal inferolateral | -10.9 [-13.4 / -9.1] | -14.5 [-18.1 / -11.8]° | -13.2 [-17.2/ -11.7] | -13.7 [-18.2 / -8.9] |
| Basal inferior | -9.6 [-13.6 / -7.7] | -11.1 [-14.7 / -8.1] | -12.7 [-15.3 / -8.2] | -14.2 [-15.3 /-12.1] |
| Basal inferoseptal | -8.9 [-10.8 / -6.3] | -9.2 [-12.7 / - 6.8] | -9.7 [-12.4 / -6.7] | -12.1 [-14.4 / -10.3] |
| Basal anteroseptal | -9.8 [-11.8 / -5.2] | -12.7 [-15.5 / -8.0]° | -11.4 [-15.1 / -7.3] | -9.9 [-14.4 / - 9.2] |
| Mid anterior | -19.8 [-21.8 / -18.3] | -19.9 [-23.4 / -17.1] | -20.2 [-24 .1 / -17.4] | -18 [-20.3 / -15.7] |
| Mid anterolateral | -20.5 [-22.7 / -16.8] | -21.0 [-22.9 / -17.6] | -20.0 [-21.6 /-17.2] | -19.6 [-21.8 / -16] |
| Mid inferolateral | -19.8 [-23.2 / -16.2] | -20.3 [-22.6 / -16.5] | -19.0 [-21.3 / -16.5] | -20.3 [-22 / -17.2] |
| Mid inferior | -19.5 [-21.8 / -16.7] | -18.9 [-21.0 / -15.9] | -18.5 [-20.6 / -15.7] | -17.2 [-20.9 / -15.8] |
| Mid inferoseptal | -16.0 [-19.0/ -13.3] | -16.3 [-20.6 / -14.1] | -15.5 [-18.2 / -12.6] | -15.8 [-18 / -14.5] |
| Mid anteroseptal | -16.5 [-18.6 / -14.5] | -16.5 [-19.3 / -13.6] | -15.7 [-18.9 / -13.5] | -13.4 [-15.6 / -11.8] |
| Apical anterior | -13.3 [-17.3 / -12.1] | -14.1 [-16.8 / -11.3] | -14.4 [-17.0 / -12.2] | -13.8 [-14.9 / -13.2] |
| Apical lateral | -15.5 [-17.8 / -14.1] | -16.9 [-18.8 / -14.5] | -16.2 [-18.3 / -15.1] | -17.4 [-18.6 / -15.4] |
| Apical inferior | -17.4 [-19.2 / -14.4] | -17.5 [-20.1 / -14.8] | -17.6 [-19.9 / -15.0] | -20.3 [-22.7 / -18.1] |
| Apical septal | -15.1 [-18.1 / -12.4] | -15.5 [-18.6 / -13.5 | -16.0 [-18.2 / -11.1] | -16 [-18.4 / -13.0] |

Values expressed as medians [interquartile range].

*P Value < 0.05 versus controls. ** P Value < 0.01 versus controls.

°P Value <0.05 versus controls after Holm-Bonferroni correction.

MVP: mitral valve prolapse; MR: mitral regurgitation

LV: left ventricle; MR: mitral regurgitation; MVP: mitral valve prolapse.
