# Supplementary material for: Mitral valve prolapse morphofunctional features by cardiovascular magnetic resonance: more than just a valvular disease
Source: J Cardiovasc Magn Reson. 2021 Oct 11;23:107. doi: 10.1186/s12968-021-00800-w (PMC8504058; doi:10.1186/s12968-021-00800-w)
Supplement: Supplementary file 5 — Additional file 5. Left ventricle peak systolic circumferential strain. Table including individual LV segments peak systolic circumferential strain measurements. [file 12968_2021_800_MOESM5_ESM.docx]

**Additional file 5. Left ventricle peak systolic circumferential strain.**

| LV peak systolic circumferential (strain %) | | | | |
| --- | --- | --- | --- | --- |
|  | Controls  (n=43) | MVP (all)  (n=78) | MVP (no significant MR)  (n=34) | “Borderline” MVP  [n=11] |
| Global | -16.4 [-17.6 / -13.8] | -16.2 [-18.5 / -14.8] | - 15.8 [-17.1 / -14.8] | -16.6 [-17.3 / -14.7] |
| Basal | -14.6 [-16 / -12.1] | -16.2 [-17.6 / -14.1]* | -15.3 [-16.8 / -14.2] | -16.6 [-18.1 / -14.7]* |
| Mid | -18.87[-21.1 / -16.3] | -18.2 [-20.5 - 16.3] | -17.5 [-19.3 / -16.4] | 17.4 [-19.6 / -16.2] |
| Apical | -16.1 [-17.9 / -14.0] | -15.9 [-18.3 /-14.0] | -15.8 [-17.7 / -13.8] | -15.9 [-18.3 / -15.2] |
| Basal anterior | -18.3 [-19.6 / -15.0] | -20.1 [-22.4/ -17.8]° | -19.6 [-22.4 / -17.6] | -17.9 [-20 / -16.3] |
| Basal anterolateral | -16.6 [-19.6 / -13.4] | -19.8 [-21.9 / -17.4]° | -19.9 [-22.5 / -17.6]° | -18.9 [-22.1 / -17.4] |
| Basal inferolateral | -15.6 [-17.7 / -11.7] | -15.4 [-17.7 /-12.7] | -15.3 [-18.2 / -13.1] | -19.5 [-22 / -15.5] |
| Basal inferior | -14.3 [-16.2 /-11.4] | -14.2 [-16.7 / -11.9] | -14.7 [-17.1 / -12.4] | -16.2 [-17.7 / -14.4] |
| Basal inferoseptal | -11.9 [-14.2/ -9.2] | -13.6 [-16.0 / -10.9] | -12.8 [-15.9 / -10.8] | -13.7 [-14.2 / -12.4] |
| Basal anteroseptal | -11.5 [-13.7 / -8.8] | -13.1 [-15.8 / -10.7] | -12.4 [-16.2 / -10.1] | -13.2 [-14.6 / -11.1] |
| Mid anterior | -19.6 [-21.2 / -17.4] | -19.8 [-22.7 / -16.0] | -20.0 [-23 / -16.2] | -17.7 [-19.8 /-14.1] |
| Mid anterolateral | -21 [-23.5 / -16.8] | -20.4 [-23.1 / -17.6] | -19.7 [-22.1 / -16.9] | -19.4 [-20.5 / -17] |
| Mid inferolateral | -20.6 [-23.3 / -17.5] | -19.7 [-22.1 / -15.9] | -19.3 [-21.1 / -15.9] | -21 [-22.1 / -17.3] |
| Mid inferior | -21.2 [-24.7 / -18.4] | -19.6 [-22.9 / -16.5] | -19.4 [-22.6 / -17.3] | -20 [-22.3 / -20] |
| Mid inferoseptal | -17.6 [-20.8 / -15.8] | -18.3 [-20.9 / -15.2] | -17.1 [-19.2 / -15.0] | -18.1 [-20.7 / -15.8] |
| Mid anteroseptal | -16.9 [-19.4 / -13.3] | -16.1 [-19.5 /-13.3] | -15.6 [-18.3 / -12.9] | -13.4 [-15.5 / -11.4] |
| Apical anterior | -14.8 [-17.9 / -12.9] | -15.5 [-17.3 / -12.2] | -14.5 [-16.8 /-12.6] | -13.6 [-14.9 / -11.4] |
| Apical lateral | -17.3 [-19.6 / -15.1] | -17.5 [-20.5 / -14.7] | -18.0 [-19.9 / -14.7] | -19.7 [-21.4 / -17.9] |
| Apical inferior | -18.1 [-20.0 / -15.3] | -18.0 [-20.9 / -15.4] | -17.7 [-20.9 / -15.3] | -20.3 [-21.4 / -17.6] |
| Apical septal | -15.2[-17.6 / -10.5] | -14.6 [-17.2 / -12.5] | -14.3 [-17.2 / -12.7] | -12.9 [-17.8 / -11.3] |

Values expressed as medians [interquartile range].

*P Value < 0.05 versus controls. ** P Value < 0.01 versus controls.

°P Value <0.05 versus controls after Holm-Bonferroni correction.

LV: left ventricle; MR: mitral regurgitation; MVP: mitral valve prolapse.
