# Supplementary material for: Mitral valve prolapse morphofunctional features by cardiovascular magnetic resonance: more than just a valvular disease
Source: J Cardiovasc Magn Reson. 2021 Oct 11;23:107. doi: 10.1186/s12968-021-00800-w (PMC8504058; doi:10.1186/s12968-021-00800-w)
Supplement: Supplementary file 6 — Additional file 6. Geometric and myocardial strain comparison between mitral valve prolapse patients with phase contrast in the ascending aorta and controls. Table showing the comparison between controls, MVP patients, and MVP without significant MR corroborated by phase contrast. [file 12968_2021_800_MOESM6_ESM.docx]

**Additional file 6. Myocardial strain comparison between mitral valve prolapse patients with posterior and bileaflet involvement.**

|  | LV peak systolic Longitudinal (strain %) | | LV peak systolic circumferential (strain %) | |
| --- | --- | --- | --- | --- |
|  | Posterior  (n=45) | Bileaflet  (n=32) | Posterior  (n=45) | Bileaflet  (n=32) |
| Global | -15.0 [-17.2 / -13.4] | -14.1 [-16.3 / -12.2] | - 16.5 [-18.6 / -14.9] | -15.8 [-17.8 / -14.1] |
| Basal | -14.2 [-17.2 / -12.3] | -13.5 [-17.6 / -8.8] | -16.5 [-17.6 / -14.7] | -16.0 [-17.5 / -13.2] |
| Mid | -18.9 [-21.2 / -16.4] | -17.3 [-20.1/ -15.7] | -18.5 [-22.0 / -17.1] | 17.5 [-29.2/ -15.7] |
| Apical | -15.5 [-18.6/ -13.4] | -15.8 [-17.9 / -14.0] | -16.0 [-18.4 / -14.2] | -15.7 [-18.1 / -13.9] |
| Basal anterior | -18.6 [-20.8 / -15.6] | -17.2 [-22.0/ -9.1] | -20.1 [-22.6 / -18.7] | -20.1 [-22.0 / -17.0] |
| Basal anterolateral | -17.6 [-21.0 / -12.9] | -16.5 [-20.1 / -9.6] | -20.4 [-22.2 / -17.6] | -19.4 [-21.8 / -16.6] |
| Basal inferolateral | -14.9 [-17.5 / -12.4] | -13.8 [-18.2 /-10.5] | -15.4 [-17.6 / -13.4] | -15.3 [-17.8 / -11.2] |
| Basal inferior | -11.8 [-14.4/ -8.2] | -10.6 [-15.5 / -6.5] | -14.3 [-16.7 / -12.4] | -13.7 [-16.5 / -11.3] |
| Basal inferoseptal | -8.6[-12.4/ -6.7] | -10.1 [-13.4/ -7.7] | -12.8 [-15.2 / -10.8] | -14.3 [-16.5 / -11.0] |
| Basal anteroseptal | -13.1 [-15.7 / -9.5] | -11.4 [-15.1 / -8.4] | -13.1 [-16.2 / -10.6] | -13.3 [-15.6 / -10.9] |
| Mid anterior | -20.6 [-23.5 / -17.9] | -18.2 [-22.1 / -15.9] | -20.0 [-22.9 / -17.1] | -17.7 [-22.7 /-15.4 |
| Mid anterolateral | -21.3 [-23.7 / -17.6] | -20.0 [-21.9 / -17.9] | -20.9 [-23.7 / -18.3] | -19.0 [-22.3 / -16.5] |
| Mid inferolateral | -20.9 [-23.6 / -16.8] | -19.0 [-21.1 / -16.1] | -20.7 [-23.4 / -17.1] | -18.7 [-20.9 / -15.2] |
| Mid inferior | -19.7 [-22.1 / -17.0] | -17.3 [-20.5 / -15.0] | -20.1 [-23.2 / -16.5] | -19.5 [-21.9 / -17.4] |
| Mid inferoseptal | -16.3 [-20.6 / -12.7] | -15.9 [-19.2 / -14.8] | -17.9 [-21.6 / -15.0] | -18.8 [-20.1 / -15.3] |
| Mid anteroseptal | -17.4 [-20.6 / -13.6] | -15.9 [-17.9 /-13.6] | -16.3 [-21.1 / -14.4] | -15.6 [-17.1 / -12.6] |
| Apical anterior | -13.8 [-16.6 / -11.5] | -14.1 [-16.9 / -11.34] | -16.2 [-17.8 /-12.2] | -14.8 [-16.9 / -12.9] |
| Apical lateral | -17.1 [-20.1 / -14.7] | -16.4 [-18.3 / -14.2] | -17.5 [-21.3 / -15.2] | -17.2 [-20.3 / -14.6] |
| Apical inferior | -17.7 [-20.2 / -14.8] | -17.4 [-19.9 / -14.8] | -17.7 [-22.0 / -14.0] | -18.4 [-20.5 / -16.1] |
| Apical septal | -15.5 [-17.6 / -13.5] | -15.4 [-18.9 / -13.5] | -14.9 [-16.4 / -12.5] | -14.0 [-16.4 / -12.5] |

Values expressed as medians [interquartile range].

*P Value < 0.05. ** P Value < 0.01.

°P Value <0.05 after Holm-Bonferroni correction.

LV: left ventricle.
